# Tiered Physician Network Plans and Patient Choices of Specialist Physicians

**DOI:** 10.1001/jamanetworkopen.2023.41836

**Published:** 2023-11-09

**Authors:** Elena Prager, Vilsa E. Curto, Alexa Magyari, Marema Gaye, Anna D. Sinaiko

**Affiliations:** 1 Simon Business School, University of Rochester, Rochester, New York; 2 National Bureau of Economic Research, Cambridge, Massachusetts; 3 Department of Health Policy and Management, Harvard T.H. Chan School of Public Health, Boston, Massachusetts; 4 PhD Program in Health Policy, University of California, Berkeley, Berkeley; 5 PhD Program in Health Policy, Harvard University, Boston, Massachusetts

## Abstract

**Importance:**

Tiered physician network (TPN) health plans sort physicians into tiers based on their cost and quality, and patients pay lower copays for visits with physicians in the lower-cost and better-quality tiers. When the plans are first introduced, they lead patients to seek care from higher-value physicians.

**Objectives:**

To examine whether TPNs are associated with patient choice of physician when the plans have been in place for 8 to 12 years and whether there are inequities in patient out-of-pocket costs associated with inequities in access to physicians in lower-copay tiers.

**Design, Setting, and Participants:**

This cross-sectional study comprising 46 645 physicians and 585 399 patients in TPNs, including 54 683 patients who had a new patient visit with a physician in a TPN, used health insurance claims data from a large employer purchaser from July 1, 2014, to June 30, 2019. Statistical analysis was performed from November 2020 to August 2023.

**Exposure:**

Evaluation and management visit with a physician in a TPN.

**Main Outcomes and Measures:**

Main outcomes were new patient market share per physician–carrier–zip code–year, distance from centroid of patient zip code to centroid of zip code of nearest low- or medium-copay physician, and mean TPN physician office visit copay per patient. A regression discontinuity design was used to estimate the association of a physician’s tier ranking, and a difference-in-differences analysis was used to estimate the association of copayment differences across tiers with market share among new patients. Equity in access was measured by comparing travel distance to the nearest physician in a low-copay or medium-copay tier and mean copayments across patient incomes.

**Results:**

The main analysis sample included 46 645 physician–carrier–zip code–year observations, 9506 (20.4%) of which were in the low-copay tier, 31 798 (68.2%) in the medium-copay tier, and 5341 (11.5%) in the high-copay tier. The 54 683 new patients in the sample had a mean (SD) age of 46.4 (16.7) years and included 33 542 women (61.3%). There was no association of having a worse tier ranking (0.045 percentage points [95% CI, −0.058 to 0.148 percentage points]) or of copayment differences between tiers (0.001 percentage points [95% CI, −0.002 to 0.004 percentage points]) with physician market share among new patients. The patients with the lowest income paid slightly lower mean (SD) copayments for office visits to a TPN physician than the patients with high income ($48.08 [$16.42] vs $51.59 [$16.79], a 6.8% difference).

**Conclusions and Relevance:**

In this cross-sectional study of TPN health plans, there was no association between physician tier ranking and physician market share among any group of patients. These findings suggest there are limitations in TPNs’ steering of patients toward high-value physicians. These plans were not associated with exacerbated health inequity in this setting.

## Key Points

**Question:**

Are tiered physician network health plans associated with patient choices of physicians over time, and do the associations vary with patient income?

**Findings:**

In this cross-sectional study of a setting where tiered physician networks have been used for a decade, there was no association between physician tier ranking and market share among any group of patients. However, patients with low income lived closer to physicians with lower copays and paid slightly lower out-of-pocket costs for tiered physician office visits than patients with high income.

**Meaning:**

This study suggests that maintaining the saliency of physician tiers for patients may be important for prolonging the benefits associated with tiered networks.

## Teaser

This cross-sectional study examines whether tiered physician networks are associated with patient choice of physician when the plans have been in place for 8 to 12 years and whether there are inequities in patient out-of-pocket costs associated with inequities in access to physicians in a lower-copay tier.

## Introduction

Patients in the US health care system value having choice among physicians. However, prices for health care services vary substantially across physicians, and price is only weakly correlated with health care quality.^1,2,3^ Thus, benefit designs that steer patients toward choosing low-cost and better-quality physicians are an important strategy in efforts to improve value. One such benefit design is tiered physician network plans, which group physicians into tiers based on a chosen definition of value and expose patients to lower out-of-pocket costs for services from physicians in higher-value tiers. Tiered physician network designs vary in how they use tiered copayments, deductibles, coinsurance, and premiums; the tiering of primary care vs specialist physicians; how physician value is measured; and whether patients select a tiered physician at the time of enrollment or at the point of care.^4,5,6,7^

Prior research on the introduction of tiered physician network plans found that they shifted patient choices away from physicians in the highest-copay tier, particularly among new patients choosing a physician for the first time.^4,8^ However, little is known about the association of tiering with patient choice of physician years after the introduction of the tiered physician network plan, when it becomes the status quo rather than a salient new intervention. Similarly, there is little evidence of how patient choices are associated with the magnitude of copay differentials across physician tiers. Finally, despite the possibility that between-tier copay differentials will matter more to patients with a lower income, there is no evidence of the equity implications of tiered physician network plans for patients with high or low income. In this study, we addressed these gaps using health care claims data from the largest employer-based health insurance purchaser in Massachusetts.

## Methods

This cross-sectional study followed the Strengthening the Reporting of Observational Studies in Epidemiology (STROBE) reporting guideline. The study was determined to be exempt by the Harvard Longwood Area institutional review board due to a category 4 exemption (secondary research for which consent is not required). A waiver of informed consent was granted by the Harvard Longwood Area institutional review board because data were deidentified.

### Study Context

Our study context was the Massachusetts Group Insurance Commission (GIC), a quasi-state agency responsible for administering benefits to employees and retirees of the Commonwealth of Massachusetts and some Massachusetts municipalities, and their dependents, in fiscal years (FYs) 2015 to 2019 (from July 1, 2014, to June 30, 2019). The GIC is the largest commercial purchaser in Massachusetts, with 8% of commercial insurance enrollment in FY2018.^9^

From FY2008 onward, the GIC included tiered physician networks in all its non-Medicare plans. Non-Medicare GIC members choose a plan once a year during open enrollment. If they then seek specialist care during the year, they select a physician from among those in their plan’s tiered network. All GIC tiered physician networks had 3 tiers, which we refer to as the low-copay (tier 1), medium-copay (tier 2), and high-copay (tier 3) tiers. During the first year of our study period, FY2015, the copayment amounts for office visits by tier were $25, $35, and $45, respectively. In FY2016, they increased to $30, $60, and $90, respectively. In FY2018, the copayment for the high-copay tier decreased to $75 in response to complaints.

### Efficiency Scores and Tier Cutoffs

Physicians were assigned to tiers on the basis of cost efficiency and quality. In practice, however, quality determined a physician’s tier assignment for fewer than 1% of tiered physicians (eMethods in Supplement 1). All other physicians were assigned to tiers based on how their efficiency scores compared with those of physicians in the same specialty.

The efficiency scores were constructed using all commercial claims from all GIC carriers, including claims from their non-GIC commercial enrollees, to avoid incentivizing physicians to treat their GIC patients differently. Symmetry Episode Treatment Groups software (Optum Inc) grouped claim lines into mutually exclusive sets of interrelated claims that formed granular “episodes of care,” such as arterial inflammation with surgery or contact dermatitis without surgery; episodes were attributed to a single physician, typically the physician whose claim lines generated the plurality of the spending within the episode (eMethods in Supplement 1). The efficiency scores measured the mean quantity of services used by a physician within an episode, controlling for patient case mix and ignoring differences in prices across physicians. For example, a surgeon who had more imaging services or longer mean lengths of stay after hip replacement surgery without complications, regardless of the surgeon’s negotiated prices with insurers or the fraction of patients receiving hip replacements, would receive a high efficiency score (eMethods in Supplement 1). A lower score indicates a more efficient physician; more efficient physicians were placed into lower-copay tiers. Because the efficiency scores deliberately ignored price differences between physicians, physicians were unable to improve their tier ranking by lowering their prices.

The GIC’s contracted health insurance carriers used these efficiency performance scores to assign approximately 20% of physicians in each specialty to the low-copay tier, approximately 65% to the medium-copay tier, and approximately 15% to the high-copay tier. Although tier assignment could vary for physicians across carriers, this was uncommon because carriers were aiming for the same size tiers.

### Data Sources

We obtained administrative enrollment and claims data for all non-Medicare GIC health plan enrollees from FY2015 to FY2019 and data on GIC employee earnings (reported in $20 000 bands to protect privacy). We merged the median annual household income for each patient’s 5-digit zip code from the 2019 American Community Survey. We obtained the physician-level quality and efficiency scores used by the carriers to assign physician tiers.

### Study Sample

We selected physicians in the 12 specialties that were tiered for a large majority (≥80%) of GIC patients: dermatology, endocrinology, gastroenterology, general surgery, neurology, noninterventional cardiology, obstetrics and gynecology, ophthalmology, orthopedics, otolaryngology, pulmonology and pulmonary disease, and rheumatology. We included physicians whose tiers were based solely on their efficiency scores and not on quality (eMethods in Supplement 1).

The patient sample included new patients who had an office visit with a physician in the physician sample. We defined a patient as new if neither the patient nor any other household member insured on the patient’s health plan had seen a physician of the same specialty in the prior 3 years. Patients with an existing relationship with a specialist likely value that relationship and, in prior research, have been shown to be unresponsive to tiering^4^; thus, we excluded households with existing physician-patient relationships. We also excluded patients who switched their health plan carrier during the study period, which avoids selection bias from patients who selected their health plan based on which physicians were in low-copay tiers.

### Outcomes

Our outcome variable was a physician’s market share among new patients. We selected each new patient’s first claim for an evaluation and management *Current Procedural Terminology* code, excluding codes for established patients (eTable 1 in Supplement 1). Then, within carrier, year, specialty, and physician zip code, we measured each physician’s market share among new patient visits in percentage points. We defined markets accounting for geographic differences in patient willingness to travel across specialties and across the state (eMethods in Supplement 1).

### Measures of Patient and Physician Characteristics

From our data, we measured patient age, sex, chronic conditions (using the Elixhauser Comorbidity Index),^10^ and years of experience with GIC tiered network plans. We measured patient income in 2 ways: the income band of the GIC employee who is the subscriber on their plan (eTable 2 in Supplement 1) and the median household income in the patient’s 5-digit zip code. We categorized physicians’ geographic locations as belonging to the Boston area if the physician’s practice zip code was within the Boston-Cambridge-Newton Core–Based Statistical Area.

### Statistical Analysis

#### Regression Discontinuity Analysis

Statistical analysis was performed from November 2020 to August 2023. Our primary analysis used a regression discontinuity (RD) study design to estimate the association of a physician’s tier ranking with new patient market share. When it is not possible to randomly assign individuals to study conditions (here, tier rankings), RD study designs are used to minimize the effect of confounding from omitted variable bias.^11^ There were likely unobserved factors associated with both patient choice of physician and physician’s tier assignment, such as physicians’ practice styles (eMethods in Supplement 1).

Regression discontinuity analyses take advantage of clinical or policy decision rules that result in assignment to an intervention on the basis of an arbitrary cutoff applied to a continuous variable.^12^ This method separates the association of the abrupt change in policy—a discontinuity—with the outcomes of interest from other factors that are also associated with outcomes, but more gradually.^11^ In our study context, some physicians with similar efficiency scores (who were very similar in use of services within episodes of care) were assigned to different tiers on the basis of whether their continuous efficiency scores were just above or just below the tier cutoffs for their specialties in a given plan year. If physicians with scores just above or just below a tier cutoff are similar, on average, then we can interpret the results of the RD analysis as measuring the association of a physician’s tier ranking with new patient market share. The validity of our RD design relies on the assumption that all other determinants of a patient’s choice of specialist physician, except for the physician’s assigned tier, would have a smooth association with efficiency scores (eMethods in Supplement 1). We tested for and did not find evidence of manipulation of scores around the tier cutoffs (eFigure 1 in Supplement 1); manipulation would also invalidate the study design.

The RD models were multivariable linear regression models in which the dependent variable was new patient market share at the physician–carrier–zip code–year level and the key independent variable of interest was an indicator for whether the physician’s efficiency score was above the cutoff for assignment to the higher-copay tier. The coefficient on this indicator measures the association of new patient market share with a physician’s assignment to a higher-copay tier. Models included controls for separate quadratic polynomials of the physician’s efficiency score (the “running” variable in the RD design) below and above the cutoff and carrier, specialty, and year fixed effects to control for unobserved, time-invariant differences across carriers and specialties and secular trends (eMethods in Supplement 1). We estimated stratified analyses by location with greater physician choice (Boston vs elsewhere in Massachusetts), restricted to obstetrics and gynecology (a specialty where patients may have more time to choose a physician), and by patient characteristics. Any reported *P* value from a regression will be for a 2-sided t-test. We used a significance cutoff of .05.

#### Difference-in-Differences Analysis of Relative Copayment Difference

To examine the association between only the relative differences in cost sharing across physicians and the physician market share of new patients, we conducted a secondary analysis using a difference-in-differences study design. The key independent variable was the difference in copayment amount between the physician’s tier and the highest-copay tier in that year, which varied due to administrative changes in copayments in FY2016 and again in FY2018. These models included physician-tier market and year fixed effects. The coefficient of this key variable measures the association between the new patient market share and the difference in copayment across tiers (ie, the monetary dimension of tiering) (eMethods in Supplement 1).

#### Analysis of Equity Implications

A potential concern with tiering is that it could create inequities in patient out-of-pocket costs for specialist visits due to inequities in access to physicians in a lower-copay tier. To examine the equity implications of the tiered physician network plans, we first examined patient access to affordable care by comparing travel distance to the nearest physician in a low-copay or medium-copay tier across patient incomes. This analysis included all GIC patients using specialist physicians, not limited to new patients. Distances are calculated between 5-digit zip code centroids; if a patient lives in a zip code in which at least 1 physician in a low-copay or medium-copay tier is also located, the calculated distance is zero. Second, we examined the mean copayments for tiered physician office visits paid across patient incomes. In these analyses, we refer to patients living in areas with median household income below $60 000, which, during our study period, was equivalent to 233% to 247% of the federal poverty level for a family of 4, as patients with low income.

## Results

### Sample Characteristics

Our main analysis sample included 46 645 physician–carrier–zip code–year observations across 12 specialties, 9506 (20.4%) of which were in the low-copay tier, 31 798 (68.2%) in the medium-copay tier, and 5341 (11.5%) in the high-copay tier (Table 1). Most (32 863 [70.5%]) practiced in the Boston area. On average, there were 387 new patient visits in a physician’s zip code within a carrier-year, and the mean physician new patient market share was 0.5%. The new patient sample of 54 683 individuals had a mean (SD) age of 46.4 (16.7) years and included 33 542 women (61.3%). The distribution of income by employee wage band is reported in eTable 2 in Supplement 1.

**Table 1.** Characteristics of Study Population

Characteristic	No. (%)
Physician tier^a^	
Low copay	9506 (20.4)
Medium copay	31 798 (68.2)
High copay	5341 (11.5)
Physician new patient market share, mean (SD), percentage points^a^	
Overall	0.47 (1.39)
Low-copay tier	0.56 (1.47)
Medium-copay tier	0.46 (1.41)
High-copay tier	0.32 (1.10)
Physician specialty^a^	
Dermatology	4037 (8.7)
Endocrinology	2121 (4.6)
Gastroenterology	5898 (12.6)
General surgery	4275 (9.2)
Neurology	2191 (4.7)
Noninterventional cardiology	3566 (7.6)
Obstetrics and gynecology	6627 (14.2)
Ophthalmology	4005 (8.6)
Orthopedic surgery	7982 (17.1)
Otolaryngology	2452 (5.3)
Pulmonary disease	2001 (4.3)
Rheumatology	1490 (3.2)
Physician practice location^a^	
Boston	32 863 (70.5)
Not Boston	13 782 (29.5)
Patient demographics^b^	
Female	33 542 (61.3)
Age, mean (SD), y	46.4 (16.7)
New subscriber (<3 y in a GIC plan)	8845 (16.18)
≥1 Chronic conditions	31 912 (58.36)
Median household income in patient’s zip code, $^b^^,^^c^	
<50 000	4725 (8.6)
50 000-59 999	5849 (10.7)
60 000-69 999	9350 (17.1)
70 000-79 999	5800 (10.6)
80 000-89 999	6808 (12.5)
90 000-99 999	6611 (12.1)
100 000-109 999	6735 (12.3)
≥110 000	8805 (16.1)
Patient insurance carrier^b^	
Fallon Community Health Plan	2431 (4.5)
Harvard Pilgrim Health Care	8585 (15.7)
Health New England	5050 (9.2)
Neighborhood Health Plan	1408 (2.6)
Tufts Health Plan	12 581 (23.0)
Unicare	24 628 (45.0)

Abbreviation: GIC, Massachusetts Group Insurance Commission.

^a^ Summary statistics for the physician sample including 46 645 physician–carrier–zip code–year observations.

^b^ Summary statistics for the 54 683 new patient visits used to construct the physician’s market share variable.

^c^ Household income is median household income in the patient’s 5-digit zip code, as reported in the 2019 American Community Survey.

### Main Results

**Figure 1.**
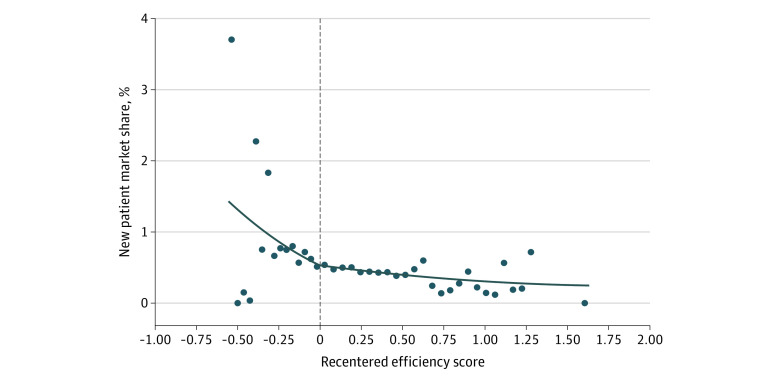
Regression Discontinuity Estimates of Association of Higher-Copay Tier With New Patient Market Share Analysis of Massachusetts Group Insurance Commission claims data and physician scores. The horizontal axis indicates the physician’s recentered efficiency score relative to the nearest cutoff score between tiers (the nearer of low-copay vs medium-copay tiers and medium-copay vs high-copay tiers). The vertical axis indicates the physician’s market share (in percentage points) among new patients. Data points indicate mean market share within bins of physicians with similar scores. The fitted line is the mean estimated market share for each score based on the regression discontinuity model. Standard errors are clustered by physician. Scores to the right of the cutoff (0 on the horizontal axis) represent physicians in higher-copay tiers. Regression discontinuity: β = 0.045 (0.053); *P* = .40; total observations included 24 187 physician–carrier–zip code–year observations.

**Figure 2.**
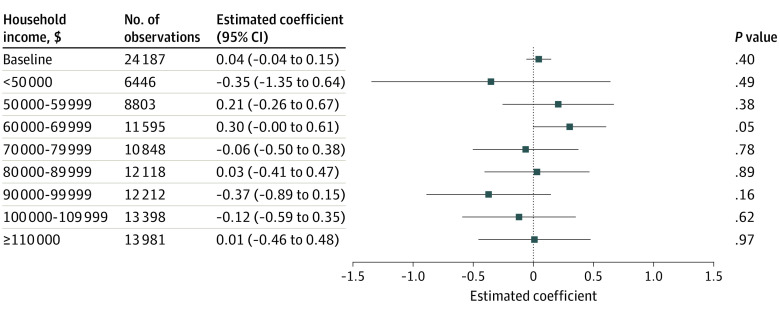
Association of Physician Assignment to a Higher-Copay Tier With New Patient Market Share by Patient Household Income, Using Regression Discontinuity Estimates Analysis of Massachusetts Group Insurance Commission claims data and physician scores. The horizontal axis indicates the estimated association in percentage points of assignment to a higher-copay tier with a physician’s market share among patients choosing a physician for the first time. Standard errors are clustered by physician. Error bars indicate 95% CIs. Estimates with error bars that did not cross the vertical line at zero were significantly different than zero. The vertical axis indicates the baseline specification (including all new patients) and the results stratified by median household income in the patient’s zip code.

**Figure 3.**
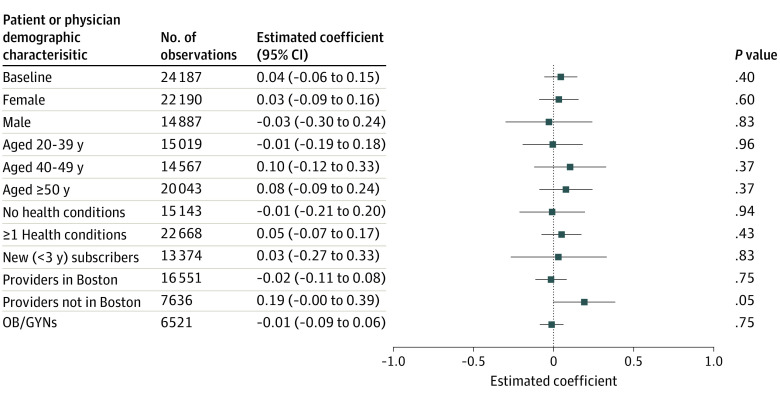
Association of Physician Assignment to a Higher-Copay Tier With New Patient Market Share by Other Physician or Patient Characteristics, Using Regression Discontinuity Estimates Authors’ analysis of Massachusetts Group Insurance Commission claims data and physician scores. The horizontal axis indicates the estimated association in percentage points of assignment to a higher-copay tier with a physician’s market share among patients choosing a physician for the first time. Standard errors are clustered by physician. Error bars indicate 95% CIs. The estimate with the error bar that did not cross the vertical line at zero was significantly different than zero. The baseline specification includes all new patients. OB/GYN indicates obstetrics and gynecology.

There was no statistically significant association between physician tier ranking and a physician’s new patient market share. We found no discontinuous change in new patient market share when the efficiency score crossed the cutoff from lower-copay to higher-copay tiers (Figure 1). This visual result is supported by the RD analysis, where the coefficient measuring the association between having a worse tier ranking and physician market share among new patients was not statistically significantly different from zero (0.045 percentage points [95% CI, −0.058 to 0.148 percentage points]) (eTable 3 in Supplement 1). There were also no statistically significant associations between physician tier ranking and new patient market share in any subgroups of patients stratified by income (Figure 2; eFigure 2 in Supplement 1), by other characteristics, by geography, or by type of physician (Figure 3).

### Relative Copayment Differences

There was no statistically significant association between a physician’s copayment relative to the high-copay tier and new patient market share in the difference-in-differences analysis. The key coefficient was 0.001 percentage points (95% CI, −0.002 to 0.004 percentage points; *P* = .57). For physicians with the modal copayment difference in our sample, $30, this is equivalent to a change in the market share of new patients of 2.9 percentage points, but this estimate is not statistically significantly different from zero. There were also no statistically significant associations between copayment differences and new patient market share in any subgroups of patients, across geographies, or by type of physician (eFigure 3 in Supplement 1).

### Equity Implications

The travel distance to the nearest physician in a low-copay or medium-copay tier was lowest for patients with low income (mean [SD] distance, 2.6 [3.9] km); it increased with increasing income and then decreased slightly. However, for most specialties, some patients with low income (10%-15%) lived farther from a physician in a low-copay or medium-copay tier than other patients (eFigure 4 in Supplement 1). Patients with low income had the greatest share of specialist visits with physicians in low-copay or medium-copay tiers (4494 of 4725 [95.1%]); this share decreased monotonically with income (Table 2). Patients in zip codes with median household incomes below $50 000 paid a mean (SD) copayment of $48.08 ($16.42) for specialist visits, which was $3.51 (6.8%) lower than that ($51.59 [$16.79]) paid by patients in zip codes with incomes of $110 000 or more.

**Table 2.** Access to Low- and Medium-Copay Tiers by Patient Income, Fiscal Years 2015-2019^a^

Patient household income, $	New patients, new patient visits		All patients, distance to nearest low- or medium-copay physician	
	No./total No. (share) of visits in the low- or medium-copay tier	Copay paid, mean (SD), $	Distance, mean (SD), km	Distance, median (IQR), km
<50 000	4494/4725 (95.1)	48.08 (16.42)	2.6 (3.9)	2.3 (0.0-3.8)
50 000-59 999	5437/5849 (93.0)	49.67 (16.82)	3.3 (6.1)	0.0 (0.0-3.3)
60 000-69 999	8794/9350 (94.1)	49.36 (16.45)	4.5 (5.2)	3.3 (0.0-6.5)
70 000-79 999	5439/5800 (93.8)	50.09 (16.31)	6.6 (7.7)	4.2 (0.0-8.5)
80 000-89 999	6345/6808 (93.2)	50.45 (16.39)	5.4 (5.4)	4.4 (0.0-7.7)
90 000-99 999	6155/6611 (93.1)	50.66 (16.29)	5.0 (5.4)	3.4 (0.0-8.8)
100 000-109 999	6182/6735 (91.8)	51.20 (16.47)	4.9 (4.7)	4.8 (0.0-8.1)
≥110 000	7965/8805 (90.5)	51.59 (16.79)	4.4 (3.9)	4.2 (0.0-7.2)

^a^ Fiscal years run from July 1 of the prior year through June 30. The patient household income is the median household income in the patient’s 5-digit zip code, as reported in the 2019 American Community Survey. Distances are calculated between 5-digit zip code centroids; if a patient lives in a zip code in which at least 1 physician in a low-copay or medium-copay tier is also located, the calculated distance is zero.

## Discussion

Tiered physician network plans may improve value in health care if they successfully steer patients to low-price or high-quality physicians.^13^ However, in the context of tiered physician network plans that had been offered through a large employer in Massachusetts for a decade, we found that a physician’s tier assignment had no association with their market share of new patient visits.

This finding is in contrast with prior studies, which found that tiered networks lead patients to choose physicians and hospitals in lower-copay tiers and that these plans are associated with lower prices and spending.^4,5,14,15^ This discrepancy may be because our study examined mature tiered networks (the 8th through 12th years of these plans), whereas prior analyses examined early periods (ie, the first through third years) when tiering may have been more salient due to the network being new and prominently advertised. It may be because the GIC’s implementation of tiered networks differed from other tiered network designs on dimensions such as copayments, premiums, and physician choice at the point of care. Changes in physician consolidation over the past decade may have changed referral patterns in a way that may have been associated with tiering.

Understanding whether any of these mechanisms explain our results would improve payers’ ability to implement tiered networks so they remain a viable tool to improve value in health care. For example, if changes in the health care delivery chain negate the strength of the tiering incentives, payers may want to focus tiering upstream in health care delivery when patients are choosing a health system in which to receive care. In Minnesota’s state employee health plans, patients are required to choose among tiered primary care clinics at the time of plan enrollment; these plans may have influenced patient choices and led clinics to lower prices for care.^7^

Tiered physician networks could be associated with health care disparities if the physicians serving lower-income communities are overrepresented in the high-copay tier. We found the opposite was true in our setting. Most patients with low income lived closer to physicians in low-copay and medium-copay tiers than did patients with high income. We also found that patients with lower income paid lower copays, on average. This result is not necessarily generalizable to other settings. In our setting, patients with lower income paid less not because the tiering steered them to physicians with lower copays but because the physicians in the lower-copay tiers were disproportionately the same physicians who already served patients with low income. In addition, our analysis of equity did not account for the role of employee premium contributions. Because employees pay the same monthly premiums regardless of income (eMethods in Supplement 1), employees with lower income are providing some subsidy to employees with higher income who choose physicians in a higher tier and receive higher quantities of services per episode of care.

### Limitations

Our analyses had several limitations. The setting for our study was a single large purchaser, which is just 1 subtype of tiered network plan designs, which limits generalizability. However, the GIC is a large employer with employees and dependents who are diverse with respect to age, income, area of residence, and job type. We only observed imperfect proxies for patient income and location. We could not be certain that patients whom we classified as new patients did not have a relationship with a specialist prior to our 3-year lookback period. Primary care physicians often influence their patients’ choice of specialist physician, and our study could not decompose results into the separate effects of tiered networks on patients vs on their primary care physicians.

## Conclusions

Tiered physician networks aim to steer patients toward choosing low-cost physicians, ideally without sacrificing quality, and are used to promote competition and value in health care markets. The findings of our cross-sectional study suggest that there are important limitations in the ability of tiered network plans to steer patients away from the lowest-quality and highest-cost physicians. Understanding how to keep tiers salient for patients in these plans will be important for extending the longevity of the benefits associated with tiered networks.

## Notes

Supplement 1.
eMethods.
**eTable 1.** Evaluation and management (E&M) codes used to select study sample **eTable 2.** Distribution of active state employees by salary band, FY2015-2019 **eTable 3.** Association of physician assignment to higher-copay tier with new patient market share, regression discontinuity design **eFigure 1.** Distribution of efficiency scores relative to tier cutoffs **eFigure 2.** Association of assignment to higher-copay tier with new patient market share using regression discontinuity, by salary of primary subscriber **eFigure 3.** Association of difference between physician’s copayment and highest tiered copayment with new patient market share, difference-in-difference analysis **eFigure 4.** Travel distance to nearest low- or medium-copay physician, by household income and specialty

Supplement 2.
Data Sharing Statement
