# Supplementary material for: Tiered Physician Network Plans and Patient Choices of Specialist Physicians
Source: JAMA Netw Open. 2023 Nov 9;6(11):e2341836. doi: 10.1001/jamanetworkopen.2023.41836 (PMC10636632; doi:10.1001/jamanetworkopen.2023.41836)
Supplement: Supplement 1. — eMethods. eTable 1. Evaluation and management (E&M) codes used to select study sample eTable 2. Distribution of active state employees by salary band, FY2015-2019 eTable 3. Association of physician assignment to higher-copay tier with new patient market share, regression discontinuity design eFigure 1. Distribution of efficiency scores relative to tier cutoffs eFigure 2. Association of assignment to higher-copay tier with new patient market share using regression discontinuity, by salary of primary subscriber eFigure 3. Association of difference between physician’s copayment and highest tiered copayment with new patient market share, difference-in-difference analysis eFigure 4. Travel distance to nearest low- or medium-copay physician, by household income and specialty [file jamanetwopen-e2341836-s001.pdf]

Tiered Physician Network Plans and Patient Choices of Specialist Physicians

Supplementary Online Content

eMethods

eTable 1. Evaluation and management (E&M) codes used to select study sample

eTable 2. Distribution of active state employees by salary band, FY2015-2019

eTable 3. Association of physician assignment to higher-copay tier with new patient market share, regression discontinuity design

eFigure 1. Distribution of efficiency scores relative to tier cutoffs

eFigure 2. Association of assignment to higher-copay tier with new patient market share using regression discontinuity, by salary of primary subscriber

eFigure 3. Association of difference between physician's copayment and highest tiered copayment with new patient market share, difference-in-difference analysis

eFigure 4. Travel distance to nearest low- or medium-copay physician, by household income and specialty

eMethods

Research overview

We used the largest employer-based purchaser of health insurance in Massachusetts to study the impacts of tiered physician networks on patient choices of specialist physicians in a setting where tiered networks have been in place for several years. The GIC tiered physician networks include tiered copayments for office visits with a specialist physician. Members paid the same deductible and had the same out-of-pocket maximum regardless of their physician's tier. About half of the plans also had tiered hospital networks, where inpatient admission copays varied with the tier of the hospital, but this was a separate tiering program. The only variation in employee premium contribution is based on employee date of hire. Members hired before FY2003 paid 20% of the monthly premium out-of-pocket. Members hired after FY2003 paid 25% of the monthly premium out-of-pocket.

If patient choices are unaffected by tiered networks, then tiered networks will not be a useful tool for encouraging patient choice of low-cost or high-quality care. Answering this question required going beyond simple comparisons of patient volumes across low-copay-tier and high-copay-tier physicians, because physician tier ranking is correlated with other physician characteristics preferred by patients. We therefore used regression discontinuity design, an analytical approach that identified the association of a physician's tier rankings with study outcomes. Our second research question was how the tiered physician networks differentially affected low-income patients. Even if tier rankings did not affect patient choices, tiered physician

networks could still exacerbate health care disparities if the physicians serving these communities were disproportionately likely to be in the least favorable, highest-copay tier. We therefore examined the distribution of tier rankings within specialty across patient incomes.

Details of GIC Scoring and Tiering Methodology

Physicians in tiered specialties were assigned to one of three tiers in each health plan in which they were in-network based on performance on a set of quality and cost-efficiency measures. Within each plan-specialty-year, the majority of physicians—approximately 65 percent of each specialty—were assigned to the medium-copay tier (Tier 2). Another 20 percent or so were assigned to the low-copay tier (Tier 1), leaving the remaining approximately 15 percent to be assigned to the high-copay tier (Tier 3). However, each carrier set the performance cutoffs for each tier, resulting in slight differences in the cutoffs across carriers. Physicians' efficiency and quality scores were calculated using all commercial claims from participating insurers, including claims from the insurers' non-GIC plans; that is, they were calculated using the insurers' full books of business. This method increased the sample size for a physician's score calculations, while avoiding incentivizing physicians to treat their GIC patients differently from other commercially insured patients.

Efficiency scores.

Physician efficiency was a summary measure of the per-episode quantity and complexity of care a physician's patients received, separate from heterogeneity in physician prices. The measure was case-mix adjusted, meaning physicians' efficiency scores were adjusted to compare

observably similar patients. More specifically, medical claims were binned into episodes using Optum's proprietary Symmetry Episode Treatment Groups (ETG) software. This software grouped together claim lines into mutually exclusive sets of relatively self-contained, interrelated claims. It aimed to attribute follow-on care and care due to complications to the same episode as the original health care service. For example, all health care services received for surgical removal of kidney stones for a patient without other comorbidities, including follow-up care, were grouped into a single episode of care.

Each episode was attributed to a single physician, typically the physician whose claim lines generate the plurality of the spending within the episode. Importantly for episode attribution, the GIC uses a unique physician identifier that can be linked across GIC insurers and over time, constructed as part of the data infrastructure supporting the GIC's tiering efforts. This physician identifier is constructed from a range of information including name, practice location, and license information. The result is more reliable for uniquely identifying physicians than tax IDs or National Provider Identifiers (NPIs) because it is never shared with other physicians in the same practice.

Each physician's efficiency for each episode type was then calculated as the average quantity of services provided under that episode type (adjusted for patient severity) multiplied by a standardized price schedule regardless of the identity of the physician. This price standardization abstracted away from the differences in negotiated prices across physicians and compared

physicians by treatment styles. As a result, physicians were unable to change their efficiency score, or their tier-ranking, by changing negotiated prices.

Episode types were fairly granular; for example, surgical removal of kidney stones for a patient without other comorbidities was a separate episode type from surgical removal of kidney stones for a patient with comorbidities. Each physician's final efficiency score used for tiering was equal to the average of her efficiency performance across episode types (weighted by episode type volume).

Quality scores.

Physician quality was defined as percent compliance with prespecified clinical guidelines. Specifically, quality was measured as the fraction of a physician's patients with a given medical condition for whom the guidelines are followed. Higher compliance rates translated to better quality scores. For example, endocrinologists were assessed primarily on quality measures related to diabetes care. A compliance rate was calculated for each physician- quality measure pair. Each physician's final quality score used for tiering was the weighted average of the relative compliance rates across quality measures (weighted by number of patients per measure).

How efficiency and quality scores were used in tiering.

In principle, tier assignments were based on a combination of quality of care and resource efficiency (also described as intensity). In practice, tiers depended primarily on efficiency, but a small number of physicians with sufficiently low quality scores were always assigned to the high-copay tier. More specifically, physicians were placed into one of three quality groupings as a function of their quality score and the statistical confidence in that score. A small number of

physicians with the very lowest quality scores were always assigned to the high-copay tier. For the majority of other physicians, quality score did not influence tier assignments with two exceptions. First, a small fraction of physicians with top quality scores who were placed in the best quality grouping realized an improvement in their final tiers. Specifically, physicians in the top quality grouping who missed the 20th percentile efficiency cutoff for the low-copay tier but who were under the 30th percentile of efficiency within their specialty were moved from the medium-copay tier to the low-copay tier. Second, physicians below the 10th percentile of quality within their specialty were at risk of reassignment to the high-copay tier regardless of their efficiency scores. We excluded physicians whose quality score had the potential to impact their tier eligibility in these ways by dropping the top and bottom quality groupings. This reduced the sample of physician-carrier-zip code-year observations by 19.21%.

Physicians whose scores were missing or based on fewer than 30 observations were placed by default in the medium-copay tier. In our sample period, all GIC insurers used the same set of quality and efficiency measures to assign physicians to tiers. As a result, physicians' tier assignments were nearly identical across insurers. This motivated our analytical approach of comparing physicians on either side of a tier cutoff within an insurer rather than comparing within a physician across insurers as in Sinaiko and Rosenthal (2014).[1]

The GIC required carriers to tier physicians based on these individual physician efficiency and quality scores until the start of FY2018. Starting in FY2018, two carriers began to use group- level tiering, whereby all physicians practicing in the same practice location within the same specialty were assigned the same tier. We dropped data from those two carriers starting in FY2018 because

our regression discontinuity design is not valid under group-level tiering. In FY2015 only, one of the GIC carriers (Fallon) only used tiering for the larger of its two GIC plans. We retained in the sample both of Fallon's plans for all years; the FY2015 observations from the non-tiered plan contributed only 0.27% of the new patient office visits in our sample.

Data linkage and defining physicians' geographic markets

To account for different patient densities across geographic markets, we measured each physician's market share only among patients who resided within a reasonable distance of the physician's primary practice location. We linked the claims data to physician data using a unique physician identifier and specialty designation that is constructed by the GIC for the purpose of tiering physicians and designed to be consistent across carriers, across physician practice locations, and over time.

To arrive at the definition of a reasonable distance among all patients treated by each specialty, we constructed the distribution of distances between the practice location zip code and patient residence zip code. We then measured the 90th percentile of the distances traveled for care within each specialty. Finally, we defined each physician's potential patient market as all patients who saw a physician in that specialty and lived within a distance no farther than the 90th percentile of distances traveled. We then calculated each physician's market share as their number of new patients in the specialty-plan-year within the physician's zip code, divided by the total number of new patients in the specialty-plan-year within the physician's zip code. Some physicians practiced in multiple zip codes and had multiple market share measures.

Regression Discontinuity (RD) design

We used a regression discontinuity (RD) study design to assess the association of tier ranking with physicians' market shares among new patients. Regression discontinuity analyses take advantage of clinical or policy decision rules that result in assignment to an intervention on the basis of an arbitrary cutoff for a continuous variable.[2,3] The validity of our RD design relies on the assumption that all other determinants of a patient's choice of specialist physician, except for the physician's assigned tier, would have a smooth relationship with efficiency scores if not for the arbitrary cutoffs.

The RD study design addresses omitted variable bias that would arise in a simple regression model. There were likely unobserved factors that drove both patient choice of physician and physician's tier assignment, for example, a physician's practice style. For example, some new patients may have avoided physicians who frequently recommend surgery over medical management. These physicians would also have had worse efficiency scores and would have been more likely to be in a higher-copay tier. Using a simple regression of market share on tier would therefore overstate the loss of patient volume attributable to being in the higher-copay tier, since it would not account for some new patients' underlying preferences to avoid the surgery-recommending physicians.

To implement the RD analyses, we focused on a subset of physicians whose tier was quasi-randomly assigned. GIC carriers determined tier rankings by first checking whether a physician passed a minimum quality threshold, and if so, then assigning a tier ranking depending on whether

the physician's efficiency score fell above or below a specialty-plan-year specific cutoff. While there were meaningful differences across physicians that may have affected patient choices, physicians whose efficiency score placed them just to either side of the cutoff between the low- and medium-copay tiers or the cutoff between the medium- and high-copay tiers should not have had discontinuous jumps in any other attributes that would impact a patient's choice to see them for care. There was also no reason to expect any other underlying differences in care provided by physicians on either side of these cutoffs other than those stemming from the impact of the tier ranking.

We checked the underlying assumptions of the RD design using widely accepted methods for RD validation.[4] First, to facilitate comparisons only between physicians whose efficiency scores were similar, we used narrow bandwidths to subset to physicians whose scores are close to the cutoff. We used data-driven mean squared error-optimal bandwidths for our primary specification, and we tested the sensitivity of our results to other choices of the bandwidths. We also used density tests to check for discontinuous jumps in the distribution of physicians' continuous scores or their attributes on either side of the tier cutoffs. Finally, we tested the sensitivity of our results to different choices of polynomial degrees.

Our main analyses were estimated using the following linear regression:

$$New\ Patient\ Share_{it} = \beta^{RD} \cdot I\{score_{it} - c_T > 0\} + f(score_{it} - c_T) + \gamma_{s(i)t} + \varepsilon_{it}$$

The outcome variable, $New\ Patient\ Share_{it}$, measured physician i's share in year t of all GIC patients seeing a physician in that specialty for the first time. This new patient share variable

was measured in percentage points, on a scale of 0 to 100. The explanatory variable of interest, $I\{score_{it} - c_T > 0\}$, was defined as an indicator for whether physician i's score in year t, $score_{it}$, was above the cutoff, c_T, for being assigned to a higher-copay tier. Its coefficient, β^{RD}, measured whether and how physician market share changed discontinuously due to being assigned to a higher-copay tier. In the language of RD analysis, the variable $(score_{it} - c_T)$ was the running variable. The linear regression controlled for $f(score_{it} - c_T)$, a flexible polynomial that captured the underlying relationship between $score_{it} - c_T$ and physician market share. In our preferred specification, $f(score_{it} - c_T)$ was defined as a quadratic polynomial and we allowed for different polynomial coefficients below and above the cutoff c_T. Recent econometric research has shown that higher-order polynomials produce unreliable estimates;[5] we did not include higher-order polynomial terms. We clustered standard errors at the level of the physician.

The term $\gamma_{s(i)t}$ represented a vector of specialty fixed effects. We included the specialty fixed effects to flexibly account for different average market shares within each specialty $s(i)$. For example, patients were divided among a smaller number of physicians in small specialties such as dermatology, compared to larger specialties such as obstetrics and gynecology (OB/GYN). On average, individual dermatologists therefore had higher market shares than individual OB/GYN physicians. We defined each physician's specialty as the specialty attributed to the physician by the GIC for tiering purposes.

In our preferred specification, we estimated this linear regression by pooling all physicians, regardless of which tier ranking cutoff is most relevant to them, and we refer to this in the paper as "Baseline." For each physician included in the pooled regression, we measured

the difference between the physician's efficiency score and the nearest tier cutoff. For example, in a specialty where an efficiency score of 0.7 or lower was required for the low-copay tier and a score of 1.4 or higher was required for the high-copay tier, a physician with a score of 0.68 would be closer to the cutoff between the low-copay and medium-copay tiers and would be coded as having a difference of -0.02 points to the cutoff with a negative value to indicate being on the low-copay side of the cutoff.

Relative copayment differences: Difference-in-Differences design

Our secondary analysis used a difference-in-differences design with continuous treatment to examine the association between copayment amounts (the monetary dimension of tiering) and physicians' new patient market share. This analysis leveraged variation in copayments within-physician due to administrative changes in copayments in FY2016 and FY2018. These analyses were estimated using the following linear regression:

$$New\ Patient\ Share_{it} = \beta^{DD} \cdot CopayDiff_{it} + \gamma_i + \tau_t + \varepsilon_{it}$$

The outcome variable, $New\ Patient\ Share_{it}$, was defined as in the RD analysis. The explanatory variable of interest, $CopayDiff_{it}$, was defined as the difference between the high-copay (tier 3) copayment amount in year t and the physician i's copayment amount in year t. (In just the subset analysis using only the subsample of physicians who were in tier 3, this variable is redefined relative to the lowest-copay tier's copayment amount.) If $\beta^{DD} > 0$, then new patient market share is higher when a physician's copayment amount is much smaller than the high-copay tier's copayment amount; in other words, new patient market share is higher when patients can save more money by choosing that physician. The physician fixed effects, γ_i,

ensured that β^{DD} was estimated by making only within-physician, within-tier comparisons to isolate the effect of changing copayment amounts. These fixed effects were defined at the granular level of physician-tier-carrier cells, but we use the γ_i notation as a shorthand. These fixed effects controlled for persistent differences across physicians in their practice styles, bedside manners, and other persistent characteristics. These difference-in-differences analyses thus measure the relationship between new patient market share and the financial differences patients face across tiers.

References

1. Sinaiko AD, Rosenthal MB. The impact of tiered physician networks on patient choices. Health Services Research. 2014;49(4):1348-1363.

2. Venkataramani AS, Bor J, Jena AB. Regression discontinuity designs in healthcare research. BMJ (Online). 2016;352:1-13.

3. Maciejewski ML, Basu A. Regression Discontinuity Design. JAMA. 2020;324(4):381. doi:10.1001/jama.2020.3822

4. Angrist JD, Pischke JS. Mostly Harmless Econometrics: An Empiricist's Companion. Princeton University Press; 2009.

5. Gelman A, Imbens G. Why High-Order Polynomials Should Not Be Used in Regression Discontinuity Designs. Journal of Business and Economic Statistics. 2019;37(3):447-456.

eTable 1. Evaluation and management (E&M) codes used to select study sample

99201-99205
99241-99255
99354-99355
99381-99399
99400-99429

Notes: These Current Procedural Terminology (CPT) codes for Evaluation & Management (E&M) visits are used to select the sample of patients newly seeing a specialist within a given specialty ("new patient visits"). This excludes E&M codes for established patients.

eTable 2. Distribution of active state employees by salary band, FY2015-2019

SalaryBand	Active employees	
$0 to $19,999	1,037	1%
$20,000 to $39,999	2,250	3%
$40,000 to $59,999	13,655	16%
$60,000 to $79,999	25,887	30%
$80,000 to $99,999	25,067	29%
$100,000 to $119,999	11,263	13%
$120,000 to $139,999	3,700	4%
$140,000 to $159,999	1,649	2%
$160,000 to $199,999	1,234	1%
$200,000 or above	489	1%

Authors' analysis of Group Insurance Commission (GIC) primary subscriber wage data.
Notes: FY = Fiscal Year. Fiscal Years run from July 1 of the prior year through June 30 (e.g., FY2015 is July 1, 2014 – June 30, 2015).

eTable 3. Association of physician assignment to higher-copay tier with new patient market share, regression discontinuity design

	Coefficient	Standard Error	p-value
Indicator for Physician Efficiency Score Above Cutoff for Higher-Copay Tier	0.0446	0.0526	0.396
Efficiency Score	-1.2362	1.1523	0.283
Efficiency Score^2	-1.0881	4.6793	0.816
Indicator for Physician Efficiency Score Above Cutoff for Higher-Copay Tier * Efficiency Score	0.8253	1.1646	0.479
Indicator for Physician Efficiency Score Above Cutoff for Higher-Copay Tier * Efficiency Score^2	1.2481	4.6848	0.790
Specialty Indicators			
Gastroenterology	-0.1734	0.0699	0.013
General Surgery	-0.0911	0.0841	0.279
Neurology	-0.0843	0.0783	0.282
Non-Interventional Cardiology	-0.1263	0.0640	0.048
Obstetrics & Gynecology	-0.4640	0.0559	0.000
Ophthalmology	-0.1641	0.0684	0.016
Orthopedic Surgery	-0.4126	0.0625	0.000
Otolaryngology	0.0151	0.0750	0.840
Pulmonary Disease	0.1234	0.0963	0.200
Rheumatology	0.4322	0.1095	0.000
Payer Indicators			
Payer 1	-0.3928	0.0611	0.000
Payer 2	1.6023	0.1907	0.000
Payer 3	0.4407	0.0924	0.000
Payer 4	-0.3591	0.0592	0.000
Payer 5	-0.3625	0.0569	0.000
Fiscal Year			
2016	-0.0289	0.0185	0.119
2017	0.0642	0.0255	0.012
2018	-0.1004	0.0331	0.002
2019	-0.0497	0.0363	0.171
Constant	0.8585	0.0898	0.000
F-statistic	17.80		
Probability > F	0.000		
R^2	0.108		
Observations	24,187		

Notes: Dependent Variable is physician's market share (in percentage points) among new patients. These results were used to create Figure 1 in the main manuscript. Standard errors were clustered at the physician level.

eFigure 1. Distribution of efficiency scores relative to tier cutoffs

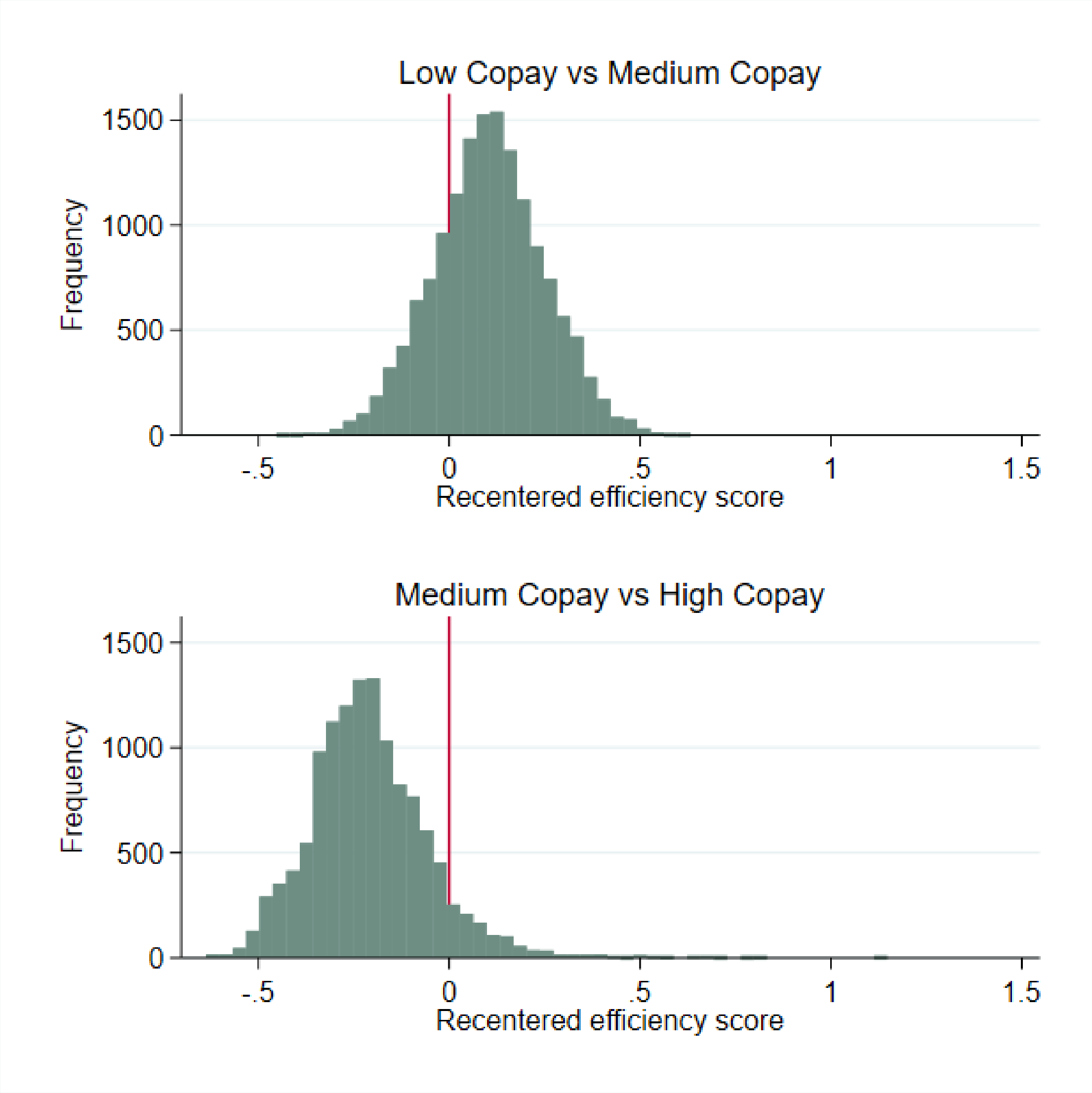

Authors' analysis of Group Insurance Commission (GIC) physician-year efficiency scores used for tier assignment.

Notes: The horizontal axis represents a physician's score relative to the cutoff score between tiers. The cutoffs between tiers vary by specialty, year, and insurance carrier. The plots are therefore normalized by subtracting the cutoff for the corresponding specialty, year, carrier, and tier pair from the physician's raw score. The top panel plots the score relative to the cutoff between the low-copay and medium-copay tiers. The bottom panel plots the score relative to the cutoff between the medium-copay and high-copay tiers. Scores to the right of the cutoff (0 on the horizontal axis) place physicians into higher-copay tiers.

eFigure 2. Association of assignment to higher-copay tier with new patient market share using regression discontinuity, by salary of primary subscriber

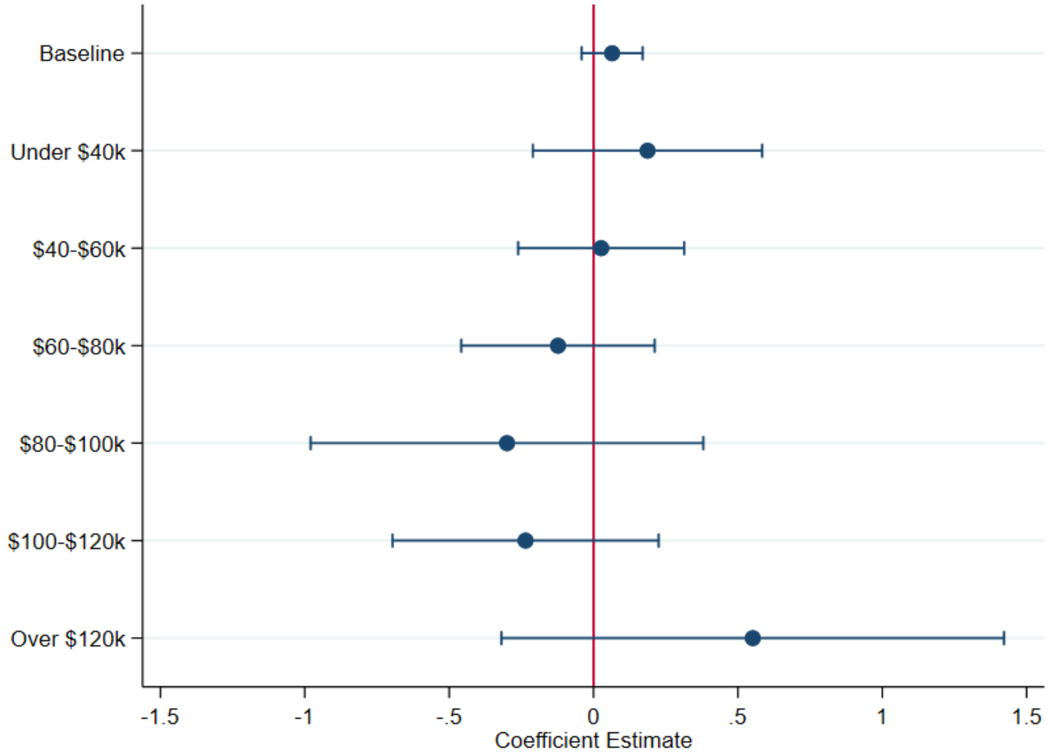

Notes: The horizontal axis shows the estimated percentage point impact of assignment to a higher-copay tier on a physician's market share among new patients. Standard errors are clustered by physician. Error bars represent 95% CIs. A negative and statistically significant coefficient would be consistent with patients being less likely to choose physicians in higher-copay tiers. Estimates with error bars that did not cross the vertical line at zero were significantly different than zero. Baseline includes all new patients.

eFigure 3. Association of difference between physician's copayment and highest tiered copayment with new patient market share, difference-in-difference analysis

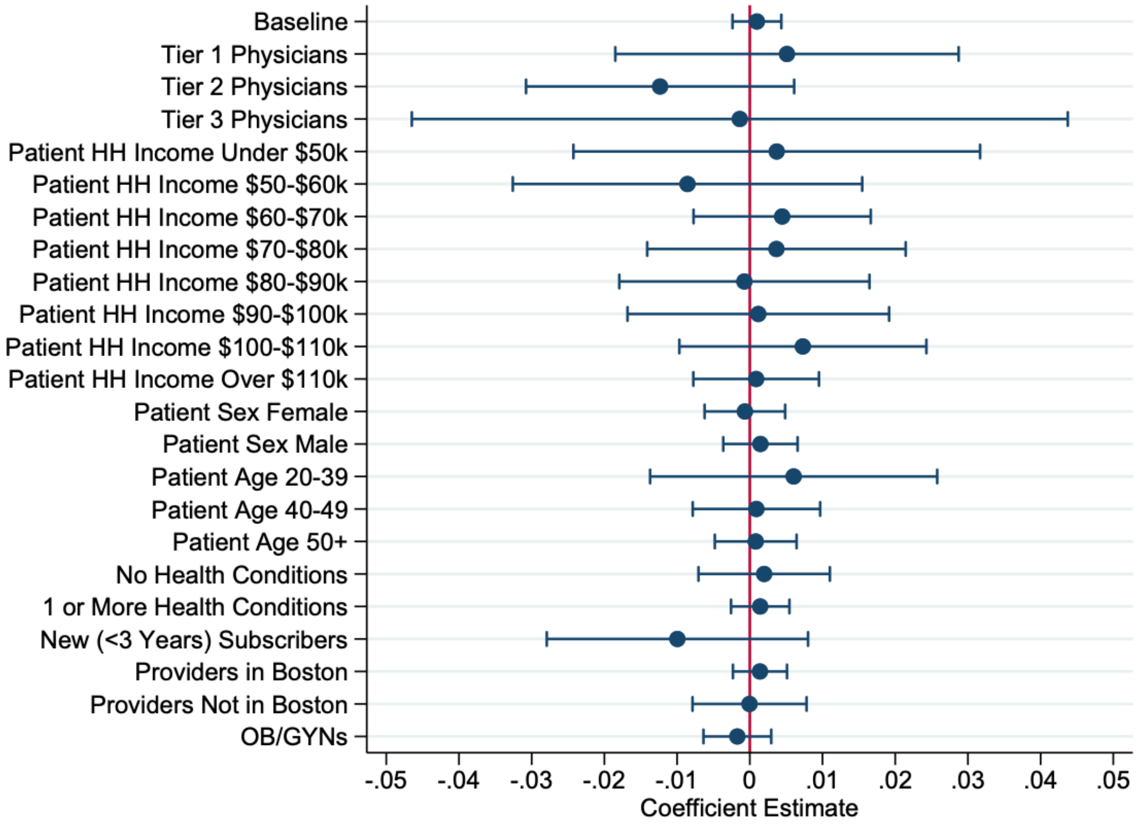

Notes: The horizontal axis shows the estimated impact in percentage points of the difference between a physician's copayment and the highest tiered copayment on a physician's market share among patients choosing a physician for the first time. Standard errors are clustered by physician. Error bars represent 95% CIs. A positive and statistically significant coefficient would be consistent with patients being less likely to choose physicians in higher-copay tiers. Estimates with error bars that did not cross the vertical line at zero were significantly different than zero.

eFigure 4. Travel distance to nearest low- or medium-copay physician, by household income and specialty

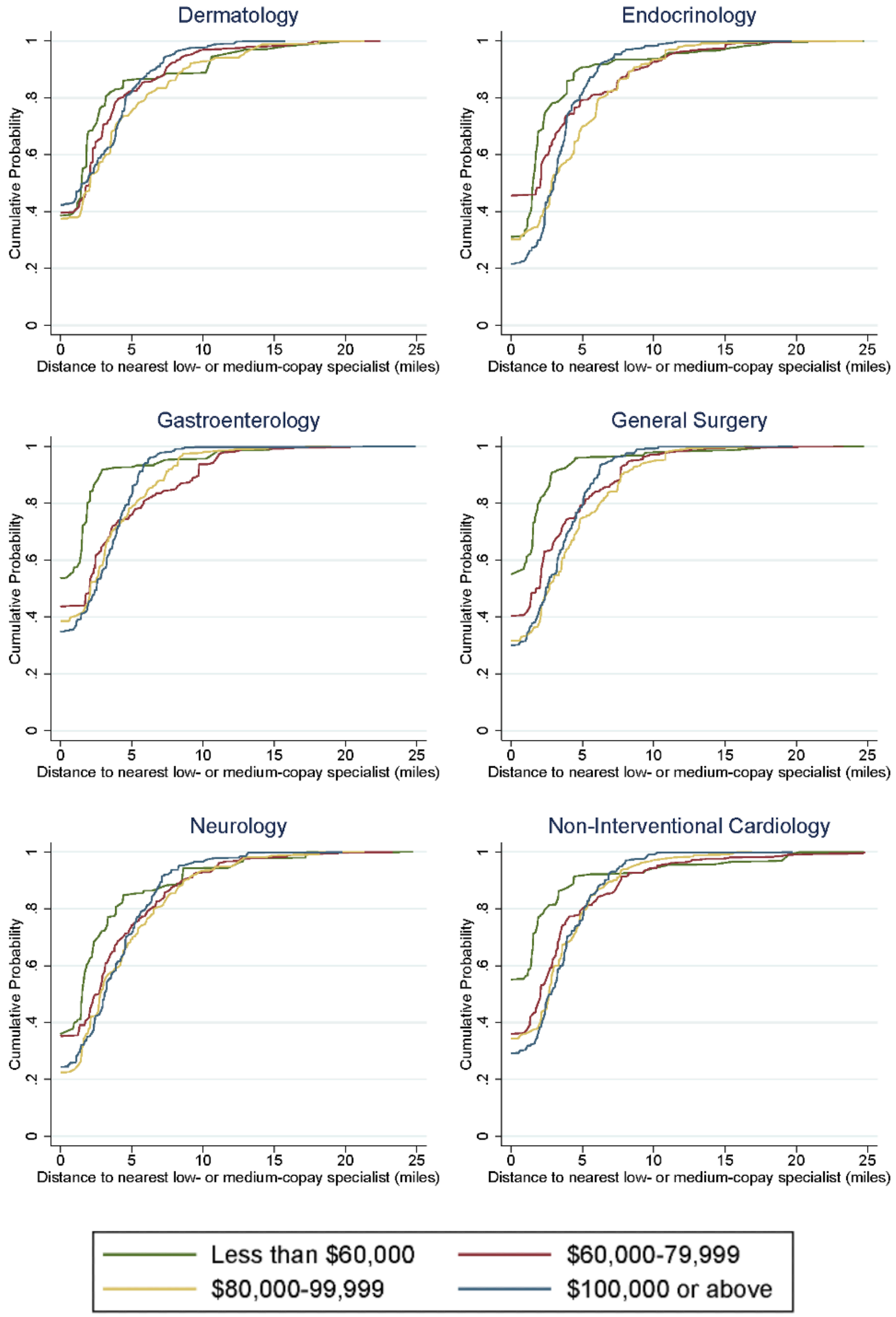

Notes: Each curve plots the share of patients of a given income range who live no farther than the plotted distance from a low- or medium-copay physician in that specialty. Income is measured as median household income in the patient's zip code. Distances are calculated between 5-digit zip code centroids; if a patient lives in a zip code in which at least one low- or medium-copay is also located, the calculated distance is zero.

eFigure 4. continued

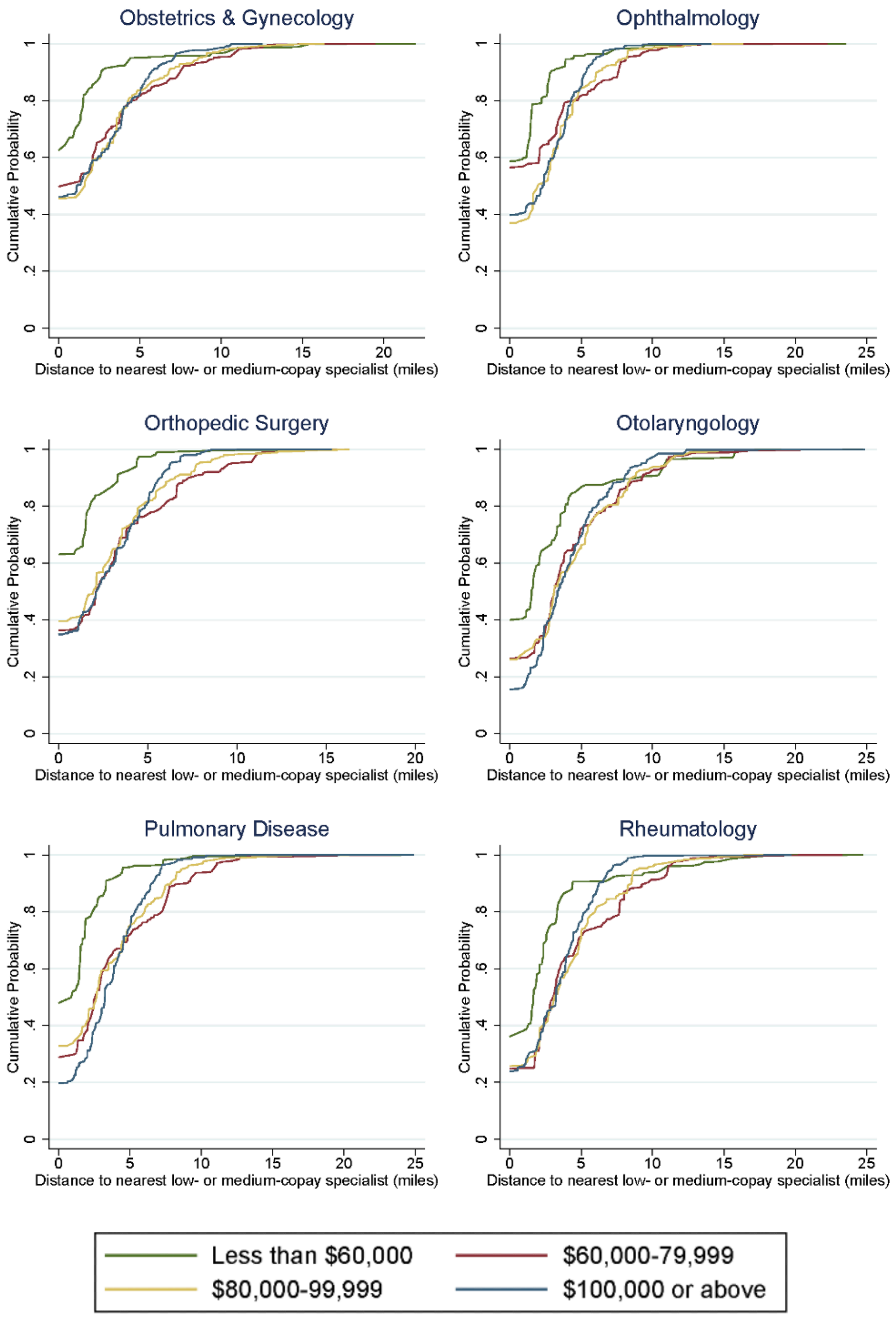

Notes: Each curve plots the share of patients of a given income range who live no farther than the plotted distance from a low- or medium-copay physician in that specialty. Income is measured as median household income in the patient's zip code. Distances are calculated between 5-digit zip code centroids; if a patient lives in a zip code in which at least one low- or medium-copay is also located, the calculated distance is zero.
