# Supplementary material for: Tiered Physician Network Plans and Patient Choices of Specialist Physicians
Source: JAMA Netw Open. 2023 Nov 9;6(11):e2341836. doi: 10.1001/jamanetworkopen.2023.41836 (PMC10636632; doi:10.1001/jamanetworkopen.2023.41836)
Supplement: Supplement 2. — Data Sharing Statement [file jamanetwopen-e2341836-s002.pdf]

Data Sharing Statement

Prager. Tiered Physician Network Plans and Patient Choices of Specialist Physicians. *JAMA Netw Open*. Published November 09, 2023. doi:10.1001/jamanetworkopen.2023.41836

Data
Data available: No

Additional Information
Explanation for why data not available: The study data are subject to Data Use Agreements which prohibit sharing of study data.
